# NTD Diagnostics for Disease Elimination: A Review

**DOI:** 10.3390/diagnostics10060375

**Published:** 2020-06-05

**Authors:** Emma Michelle Taylor

**Affiliations:** Department of Social Anthropology, University of Edinburgh, Edinburgh EH8 9LD, UK; E.M.Taylor@ed.ac.uk

**Keywords:** diagnosis, neglected tropical disease, disease elimination, point-of-care diagnostics

## Abstract

Neglected Tropical Diseases (NTDs) marked out for disease elimination provide a lens through which to explore the changing status of diagnosis in global health. This paper reports on the findings of a scoping review, which set out to explore the main debates around diagnosis for the elimination of NTDs, including the multiple roles diagnostic technologies are being ascribed and the ideal characteristics of tests. It also attempts to summarise the state of diagnosis for three NTDs with elimination goals. The review places special emphasis on point-of-care testing in acknowledgement of the remote and underserved areas where NTDs proliferate. Early NTD campaigns were largely focused on attack phase planning, whereby a similar set of interventions could be transplanted anywhere. Now, with elimination goals in sight, strategies must be tailored to local settings if they are to attain and sustain success. Diagnostic data helps with local adaptation and is increasingly used for programmatic decision-making. The review finds that elimination goals reframe whom diagnosis is for and the myriad roles diagnostics can play. The exigencies of elimination also serve to highlight deficiencies in the current diagnostic arsenal and development pipeline for many NTDs. Moving forward, a guiding framework is needed to drive research and stimulate investment in diagnosis to support NTD goals.

## 1. Introduction

The current COVID-19 crisis has brought the importance of diagnosis into sharp relief, but even prior to this, the status of diagnosis in global health has been changing. The publication of the first Essential Diagnostic List (EDL) in 2018 signalled a turning point, suggesting for the first time that diagnostics could come to occupy a comparative status in global health as pharmaceuticals [1]. Other key milestones include the launch of the Foundation for Innovative New Diagnostics (FIND), the establishment of the Global Diagnostics Forum, and the launch of the Lancet Commission on Diagnostics. In 2019, the World Health Organisation (WHO) convened a new Diagnostic Technical Advisory Group for Neglected Tropical Diseases (NTDs), and in 2020 plans to launch a revised “NTD Roadmap”, for the period 2021–2030, assigning diagnosis a pivotal role [2]. This breaks with the peripheral position afforded diagnosis in the last “NTD Roadmap” [3], which served to formalise an ambitious disease elimination and eradication agenda for many of the named diseases but largely prioritised pharmaceutical solutions.

There is hardly a better lens through which to view diagnosis’ changing status than through NTDs. Toward the beginning of the last decade, NTD campaigns were best described as a “steam roller” of drugs crossing the African continent (Prof. Alan Fenwick referring to mass drug administration at the International Society for Neglected Tropical Diseases conference on 12th February 2013). Today, with disease elimination and eradication goals in sight for many diseases, approaches have grown more nuanced and sophisticated. Prevention has been afforded a much bigger role (as demonstrated by WHO strategy papers on water, sanitation and hygiene, and vector control [4,5]). In addition, real-time data is increasingly informing programmatic decision-making, thanks to data platforms like ESPEN Collect (Expanded Special Project for Elimination of Neglected Tropical Diseases) and Tropical Data (the data-collection initiative supporting trachoma elimination) [6,7]. This data is being used to map the geographical spread of NTDs; to determine whether programmes need to scale up or scale down (or change tack); and, once disease goals are achieved, to help programmes sustain success (this list is not exhaustive). Diagnosis is playing an increasingly important role in this data revolution, with the rigours of elimination goals both reframing the myriad roles diagnostic technologies can play and highlighting deficiencies in the current diagnostic arsenal and development pipeline.

Reporting on the results of a scoping review and supporting discourse analysis, this paper attempts to synthesise the main debates around diagnosis for the elimination of NTDs, exploring the multiple roles diagnostics are being ascribed, the ideal characteristics of tests, and the state of diagnostics for three foci NTDs.

The paper boasts an express interest in field applicable or point-of-care testing. This is in recognition of the settings where NTDs predominate—where basic health infrastructure is sparse or wholly absent [8]. New point-of-care testing technologies have great potential for both improving access to care and generating high-quality epidemiological data for NTD prevalence in the under-resourced settings where NTDs are commonly found.

### NTDs and Disease Elimination

In the new millennium, the case was made that a number of tropical infections should be taken forward as a group by virtue of their “neglected” status and shared geographic overlap, and because cost savings and synergies could be levied if the different disease programmes acted together [9,10]. The resulting categorisation—Neglected Tropical Disease (NTD)—has subsequently become a successful “brand identity” [11].

The WHO helped create cohesion around NTDs by naming an initial grouping of 17 diseases in 2010 [12] and then incorporating these into a joint framework for action in 2012: the “NTD Roadmap” [3]. The Roadmap contains targets relating to the NTDs (in their previous incarnations as unconnected diseases) based on existing World Health Assembly resolutions [12] (pp. 155–157). The Roadmap served to collate these targets—which include control, elimination, and eradication goals—in one umbrella document, and together with the London Declaration on NTDs [13], mobilised action in pursuit of an overarching 2020 agenda. The revised Roadmap to be launched in 2020 will move the focus on to 2030, better aligning the NTDs with the Sustainable Development Goals [2]. While both Roadmaps have presented the NTDs as a collective grouping, it is significant that most disease programmes continue to operate in a largely vertical manner (with their own budgets and reporting systems).

Successful campaigning has resulted in increased attention and resources for NTDs. Aligning the diseases with global goals—Millennium Development Goals, Sustainable Development Goals, and disease-specific targets like disease elimination—has further motivated action [9,14,15].

The history of global health in the 20th and 21st centuries attests to widespread attraction to the concept of disease eradication. The intuitive appeal is simple: frontload investment now in order to save money on prevention and control in the future. This style of thinking took root at the Rockefeller Foundation before WWII, then was carried on after the war by the newly formed WHO, which led campaigns against yaws, yellow fever, malaria, and smallpox [16]. As an approach, however, eradication fell out of favour with the failure of the malaria eradication programme in the 1950s. Various conferences and initiatives convened in the last 20 years have served to reignite the concept (the 1997 Dahlem Workshop on the Eradication of Infectious Diseases; the 1998 Conference on Global Disease Elimination and Eradication as Public Health Strategies; the International Taskforce for Disease Eradication). Many candidates have been discounted for eradication, stimulating debate that has resulted in new categorisations of disease—namely, diseases with the potential to be eradicated in the future, and diseases which could feasibly be eliminated within a specified area in the short- to medium-term [17]. This widening of the definitional net has seen the number of diseases targeted for disease elimination swell, a trend demonstrated by the range of NTDs marked out for elimination in the 2012 “NTD Roadmap” (Table 1).

One outcome of this proliferation of targets has been to expose the terminological confusion around such goal setting. In an attempt to provide clarity around the three interrelated terms, disease eradication, elimination, and control, Molyneux, Hopkins, and Zagaria endorsed the definitions presented in Box 1.

Box 1 **Glossary of control, elimination, and eradication terminology [18].**

**Control**: Reduction of disease incidence, prevalence, morbidity or mortality to a locally acceptable level as a result of deliberate efforts. Continued intervention measures are required to maintain the reduction.

**Elimination**: Reduction to zero of the incidence of a specified disease in a defined geographical area as a result of deliberate efforts. Continued intervention measures are required.

**Eradication:** Permanent reduction to zero of the worldwide incidence of infection caused by a specific agent as a result of deliberate efforts. Intervention measures are no longer needed.

These definitions help us distinguish between “eradication” (which is always understood to be global) and “global elimination”, by pointing out that it is the continued need for intervention measures that renders elimination the less ambitious goal. That said, these definitions although helpful, somewhat belie the complexity still residing within disease-specific elimination goals. For instance, human African trypanosomiasis has been set two elimination goals of varying ambition. By 2020, the hope is that the *T.b. gambiense* strain of the disease will be eliminated “as a public health problem”, while by 2030 it is hoped that transmission of *T.b. gambiense* will be reduced to zero (see Section 3.2 for more detail). With disease elimination, therefore, scrutinising the precise wording of goals is important.

Although intuitively appealing, disease eradication is hugely challenging (hence why only one disease affecting humans, smallpox, has been successfully eradicated). The essential difficulty is that while gains in the attack phase can be achieved with relative ease and through the transplantation of a similar set of interventions anywhere, the closer you get to the endgame the more the challenges and costs escalate, and the more interventions and approaches have to be fine-tuned to local settings [16]. This is the experience of the Guinea-worm and poliomyelitis campaigns [19], as well as the context behind Dowdle and Cochi’s summation that “all eradication is ultimately local” [20] (p. 3). The central importance attached to diagnosis, as a means by which one might hope to understand and manage local settings, is now a key message coming out of the literature [21,22,23]. In this view, diagnostics and the data they yield promise to become a missing piece in the puzzle of disease elimination.

The scoping review that informs this article was conducted as part of the ERC-funded DiaDev project (www.diadev.eu, accessed on 20 May 2020). DiaDev is investigating the design and use of diagnostic devices in global health. Key questions for DiaDev’s elimination theme are: How does the goal of disease elimination inform our thinking around what, and whom, diagnosis is for? And what are perceived to be the ideal characteristics of the testing approaches required to achieve it? These questions are explored in this paper.

## 2. Materials and Methods

This paper reports on the results of a formal scoping review to look into the state of diagnostics for the NTDs assigned disease elimination goals in the 2012 “NTD Roadmap” [3]. Before attempting the review, a discourse analysis was undertaken to determine which of the 17 diseases the WHO originally termed “NTDs” in 2010 (WHO now supports 20 NTDs) had (a) specific disease elimination goals, and (b) outstanding diagnostic needs in relation to those goals [3,12]. Hence, from an initial group of 17 diseases, 6 were immediately discounted for having goals related only to disease control (dengue, buruli ulcer, cutaneous leishmaniasis, taeniasis/cysticercosis and echinococcosis/hydatidosis, foodborne trematode infections, and soil-transmitted helminthiasis), 2 were discounted for having eradication goals (Guinea-worm and yaws), and two were discounted for having no outstanding diagnostic need (dog-mediated rabies and leprosy). This left seven NTDs, which became the focus of the review: Chagas disease, human African trypanosomiasis, lymphatic filariasis, onchocerciasis, schistosomiasis, trachoma, and visceral leishmaniasis. For the purpose of this paper, I only present synthesised results “by disease” for three NTDs: human African trypanosomiasis, onchocerciasis, and schistosomiasis. These represent a mix of diseases requiring Intensive Disease Management and Mass Drug Administration (concepts explained in the Results section), whose diagnostic capabilities range from “inadequate” to “adequate” to meet 2030 disease targets in the newest edition of the “NTD Roadmap” [2] (p. 34).

The Web of Science Core Collection was searched on various dates in December 2018 and January 2019 to identify disease-specific publications that included topic hits on disease elimination and disease diagnosis. The timespan of published papers was limited to the period 1 January 2012–31 December 2018. This time period reflects the fact that, while various World Health Assembly resolutions in relation to disease elimination had been put in place prior to 2012, the publication of the WHO’s “NTD Roadmap” in January 2012 served to collate these targets in one overarching document [3] and, together with the London Declaration on NTDs [13], mobilised action in pursuit of a bigger 2020 agenda. An overview of the search strings used in the review is provided as Supplementary data.

Searches were conducted using the Web of Science’s online search tool. The results were then exported into Microsoft Excel, where each paper’s suitability to be included in the scoping review was determined according to the author’s reading of the title and abstract. In total, 3465 papers were identified, of which 448 were included in the review (a full list of included papers is included as Supplementary data). The breakdown of results by disease is presented in Table 2.

The main themes identified in the included papers were analysed using the qualitative software package NVivo. The results were presented as a narrative synthesis.

Due to limited time and resources, the search for this review was confined to a single database. It was not possible to review the abstracts for all the papers, notably conference papers. In this case, papers were “included” by default.

## 3. Results

The yield of included studies in the scoping review was subdivided into two categories: (1) papers addressing the issues facing NTD diagnosis in broad terms, i.e., presenting a global overview, and (2) papers addressing the foci NTDs marked out for elimination in some detail.

### 3.1. Global Overview

A close reading of the papers in this grouping revealed a general consensus around a number of themes and positions. Setting out whom diagnosis is targeting and the specific roles (or use cases) for diagnosis in light of elimination goals provides a useful backdrop to understand the peculiar diagnostic needs of NTD programmes. Establishing the strengths and limitations of existing testing approaches in the NTD field prefaces an exploration into some of the new technologies on the horizon and the ideal characteristics of future tests. Finally, an exploration of some of the main barriers facing innovation in the diagnostic field concludes this section.

#### 3.1.1. Setting out Whom and What Diagnosis is for in NTD Elimination Campaigns

Several of the papers subdivided the group of NTDs being targeted for elimination into two categories: (a) diseases amenable to Mass Drug Administration (MDA), (“An MDA program requires repeated distribution of treatment to large numbers of individuals, without diagnosis” (Hollingsworth 2018: s240)) and (b) diseases requiring Intensive Disease Management (IDM) [21,22,24]. This distinction is pertinent, as it changes both the unit of intervention and the ascribed role of diagnosis.

For MDA-amendable diseases like schistosomiasis, lymphatic filariasis, and onchocerciasis, the unit of intervention is the target population. These diseases require tests to identify and map populations requiring treatment and to ensure transmission is interrupted. New diagnostics are envisaged to play a critical role in monitoring progress towards elimination goals and for ongoing surveillance [21]. For these diseases, data is needed to determine when and where MDA should be delivered; then “the key questions are who to treat (e.g., which age group), how often to treat, and when treatments can be stopped” [22] (p. 241). For IDM diseases like Chagas disease and visceral leishmaniasis, the unit of intervention is the individual. For some IDM diseases, like human African trypanosomiasis (HAT), diagnosis cannot be accomplished with a single test. Instead, a diagnostic algorithm must be followed to confirm infection—which for HAT includes screening, parasitology, and disease staging [25]. There are a number of common uncertainties around IDM diseases which act to delay and complicate the pathway to diagnosis—for instance, long uncertain incubation periods and an unknown degree of transmission by asymptomatic carriers [22].

Hollingsworth suggests the current MDA/IDM dichotomy is “part of a shifting landscape that is dependent on a changing epidemiology, demography, and on the availability of new tools” [22] (p. 242). For instance, some IDM diseases could be treated with MDA if a safer drug were developed, while the programmatic approach could shift to a test-and-treat campaign (or even case management) if the right diagnostic became available for a MDA-amenable disease or if local elimination was achieved.

The traditional role of diagnosis for patient management—to rule in or out infection—is added to by the specific needs of elimination programmes [26]. For instance, Solomon et al. conceptualise four time points when MDA-amenable disease elimination programmes will require diagnosis: for mapping, for impact monitoring, to inform stop-MDA decision-making, and for post-elimination surveillance [8]. Additional use cases for diagnostics in support of elimination programmes could centre on monitoring drug efficacy and drug resistance [27].

Several of the papers in this yield paid particular attention to the role diagnostics might play in disease surveillance and response [26,28,29,30]. Zhou, Bergquist, and Tanner define the aims of a NTD “surveillance and response” system as “discovery, investigation, and elimination of continuing transmission, the prevention and cure of infection and final substantiation of claimed eradication” [28] (p. 1). They underline that a key feature of surveillance response is its focus on a set of minimum data as opposed to classical monitoring and evaluation, with its focus on collecting all possible data. Research priorities for the establishment of a successful surveillance response system for NTDs include the development of novel tools to sensitively detect low-transmission patterns and (re-) emerging pathogens.

#### 3.1.2. Existing Diagnostic Approaches for NTDs

In countries where laboratory infrastructure is limited, the WHO has advocated the use of syndromic diagnosis; and trachoma is one NTD that continues to be diagnosed clinically. As an approach, however, syndromic diagnosis often results in over diagnosis and treatment [26]. This may put a strain on limited resources, or—as is the case with trachoma—be tolerated in pursuit of disease elimination.

Microscopy continues to play a central role in the diagnosis of many NTDs, including lymphatic filariasis and schistosomiasis, and relies on the training and expertise of professionals, as well as the availability of laboratories with functioning microscopes [24,31]. While microscopy for parasites is highly specific, its sensitivity depends on the intensity of infection. As prevalence and infection intensity fall—for example in response to treatment—more sensitive and/or specific tests will be required to secure elimination [8,24,26,28,30].

To take the example of intestinal schistosomiasis (specifically *S. mansoni*), while a trained microscopist can identify a single schistosome egg in a faecal smear prepared by Kato–Katz, not all the samples from an infected person will present with eggs, particularly if the patient is suffering from a low-intensity infection. Examining multiple samples over consecutive days may be a tolerable work around in the early stages of an elimination campaign but as the elimination goal is neared, only a more sensitive test, preferably with high-throughput capabilities, will suffice. More specific tests may also be required as disease goals are met to ensure the correct pathogen is being identified. Five schistosoma species cause intestinal schistosomiasis, yet the current circulating cathodic antigen urine test is only effective at detecting *S. mansoni* (cross reactions of diagnostic tests to other pathogens is a wider concern in the literature [32,33,34,35]). A final point of clarification is that it could still be possible to better diagnose residual cases in elimination campaigns using existing diagnostics (i.e., without improving sensitivity or specificity). To manage for variations in the performance of existing tests, repeat testing, combining, and/or sequencing tests may help achieve the sensitivity and specificity required for elimination [24,31]. Equally, it could be possible to diagnose individuals with high-intensity infections in areas of low disease prevalence just by altering sampling strategies.

Immunodiagnosis is already being delivered for NTDs, with rapid immunochromatographic tests (ICTs) offering onsite results and enzyme-linked immunoassays (ELISAs) offering higher sensitivity and high-throughput capability [24]. Yet, while immunodiagnosis is available for many NTDs, existing tests may lack sensitivity and/or specificity, and while some serological tests perform well (e.g., for Chagas disease), others are error prone (e.g., certain helminth species cross-react with antigens from other helminths); moreover, their availability may vary from one setting to another [31]. Peeling summarises the issue, noting that while in the last decade point-of-care tests fulfilling the Affordable, Sensitive, Specific, User-friendly, Rapid and robust, Equipment-free and Deliverable to end-users (ASSURED) criteria have become commercially available, “the quality of these tests varies, quality of testing is often not assured and there are few mechanisms to capture test results for surveillance when the testing is so decentralised” [26] (p. 385). A new generation of immunoassays could start to address these deficiencies. For instance, bead-based immunoassays boast increased sensitivity and versatility over ELISAs, as well as the potential to detect multiple pathogens using a single specimen. Microfluidic immunoassays provide similar advantages whilst also offering short analysis times and high-throughput capabilities; they also tend to be quick and inexpensive to manufacture [24,26].

Until recently molecular tools tended to be the preserve of research groups, yet there is now hope that molecular assays could one day replace microscopy and immunodiagnosis for some NTDs [31]. The advent of several point-of-care nucleic acid amplification tests (POC NAATs) like GeneXpert has made this more likely [24]. Yet, while GeneXpert boasts many advantages—e.g., being able to test for different pathogens, requiring minimal onsite expertise, and offering quick results—it requires electricity, and without subsidisation its cartridges remain unaffordable for many low- and middle-income countries. Therefore, cost-efficient means of implementing novel technologies will need to be found if they are to maximise their potential for NTDs [26]. Already, loop-mediated amplification (LAMP) assays have been developed and evaluated for visceral leishmaniasis and human African trypanosomiasis, but as Peeling remarks, “Advocacy and investments are needed to apply these technologies to the control and elimination of NTDs” [26] (p. 387).

#### 3.1.3. Future Diagnostic Development

According to Peeling and Mabey, “The development of appropriate diagnostics starts with defining target product profiles” (TPPs) [24] (p. 1792). TPPs are a description of the ideal specifications (performance and operational) needed for a given product, considering the needs of the patient and the main characteristics of the relevant health system. To think through some examples, as MDA-amenable diseases near elimination, large number of samples will need to be tested, so high-throughput diagnostics will be desirable. Meanwhile, for IDM diseases the need will be for point-of-care tests to reach residual or (re-)emerging infections in remote and rural populations.

Many of the included papers reflect on the ideal characteristics of testing technologies to be deployed as disease prevalence and/or infection intensity fall in order to certify elimination and support post-elimination surveillance. Here again, the uniform message is that more sensitive and/or specific tests will be required [8,24,26,28,30].

A number of papers in this grouping focused on the role diagnostics might play in generating data to inform elimination programmes “to provide timely information on testing, trends, quality assurance and…to optimise supply chain management” [36] (p. 273). They point to the number of point-of-care devices now available that can digitise and transmit data, including a new generation of immunoassays and molecular technologies that can be combined with readers or mobile phones to collect data [24]. In addition to improving day-to-day programme management, such technologies have the potential to transform disease surveillance in a post-elimination context [24,26,28]. However, the management of digital data requires some forethought to question the kinds of systems, software, and IT knowledge that would be needed to collect, store, and govern it [26,36].

Starting with the premise that “It is neither feasible nor efficient for countries to manage 17 NTDs as individual programmes, each with its own range of diagnostic and surveillance tools” [36] (p. 3), the aspiration that a multiplex platform could one day be developed for NTDs is a common concern within the literature [8,36,37]. Similarities between the TPPs of seven MDA-amendable NTDs (trachoma, lymphatic filariasis, schistosomiasis, onchocerciasis, and soil-transmitted helminths) suggest that the integration of diagnostic approaches is feasible, with a multiplex platform potentially allowing mapping, treatment, impact monitoring, and post-elimination surveillance to be “coordinated to better utilise limited human and financial resources” [8] (p. 1). In considering the ideal characteristics of such a platform, Solomon et al. reflect on the contexts in which NTDs thrive: areas with little health infrastructure, varying laboratory access, and little potential for data collection (at least, without additional resourcing). They conclude that “The ideal integrated system might therefore be a portable, self-contained diagnostics platform, capable of performing multiplex assays for several infections of interest on one or a small number of sample types” [8] (p. 4). Since foci diseases vary from one population to next, a modular format would be ideal. This would have the added benefit of testing for co-infections like HIV and malaria. Lammie et al. also support multiplexing and suggest that an existing diagnostic platform using Luminex-based antibody assays could be used to integrate surveillance for NTDs with the monitoring and evaluation of other public health efforts. Yet, while one biplex test has been developed for onchocerciasis/lymphatic filariasis [38,39] and another is being explored for human African trypanosomiasis/malaria [40], the dream of a multiplex diagnostic platform for a broad spectrum of NTDs still appears some way off. One stumbling block to this vision is the lack of well-characterised and validated antigens for the monitoring of antibody responses at the population level for several of the MDA-amenable NTDs [37].

#### 3.1.4. Barriers to Unlocking the Potential of Diagnostics

Despite consensus that diagnostic tools boost great potential to support NTD elimination, the literature highlighted a number of serious challenges. The theme of neglect and its knock-on effect for diagnostic innovation is addressed at some length, with the perceived lack of a commercially viable market for diagnostics depicted as a major barrier [21,26,31,36]. This is hampered at once by a lack of public and philanthropic funding—to overcome market failure—and by a research and development pipeline that is not sufficiently robust [21]. The upshot is that “While progress has been made [for NTDs] in the last decade with chemotherapy reaching a billion people in 2014, the same cannot be said of diagnostics…” [36] (p. 271)

Peeling, Boeras, and Nkengasong acknowledge that “The barriers faced in implementing testing at point-of-care are often not technological, but constraints inherent in the health care system” [36] (p. 273). They expand on the myriad infrastructure requirements needed to support point-of-care testing. Subsequently, while it is generally accepted that the deployment of point-of-care tests can provide opportunities for health systems to be strengthened [36], the converse can also be true, with point-of-care testing exerting additional pressure on weak health systems [26].

Several papers made allusions to the hurdles involved in getting new diagnostic technologies from lab bench to end users [8,24,26]. A further two papers highlighted the “important gaps in our understanding of the epidemiology and control” of the NTDs that could yet alter the trajectories of disease programmes [22,31] (p. 17).

### 3.2. By Disease

In this section, I summarise the main diagnostic issues faced by three NTDs with elimination goals. In the new edition of the “NTD Roadmap”, WHO has presented the existing diagnostic capabilities of NTDs using a traffic light system. In this snapshot from the larger scoping review, I present the state of diagnosis for three NTDs, a mix of IDM and MDA-amenable diseases whose diagnostic capabilities are said to range from “inadequate” to “adequate” to meet their 2030 disease targets [2] (p. 34).

#### 3.2.1. Human African Trypanosomiasis

Human African Trypanosomiasis (HAT), also known as African sleeping sickness, is a parasitic disease transmitted by the tsetse fly. The disease is caused by two subspecies of the African trypanosome: *T. b. rhodesiense* and *T*. *b*. *gambiense*. Given that *T. b. rhodesiense* is zoonotic, elimination is not deemed feasible at this time. Without treatment, HAT is ordinarily fatal. Elimination goals for HAT are presented in Box 2.

Box 2 **Elimination goals for human African Trypanosomiasis.**

**Goal: Global Elimination**

By 2020: Eliminate *T. b. gambiense* as a public health problem (defined as the reduction of *gambiense* HAT incidence to less than 1 new case per 10,000 population at risk, in at least 90% of foci with fewer than 2000 cases reported globally).

By 2030: Eliminate *T. b. gambiense* transmission to zero (defined by a reduction of HAT incidence to no new cases from any foci by 2030).

The signs and symptoms of HAT are diverse and non-specific. Early diagnosis is needed to prevent the disease from progressing from stage 1 to stage 2 (the neurological phase). Given the toxicity of treatments, a confirmatory diagnosis is needed to access treatment and follows a complex algorithm involving screening, parasitological confirmation, and disease staging [25]. Until recently, the only screening tool for *T.b. gambiense* was the Card Agglutination Trypanosomiasis Test (CATT), an antibody test first developed in the 1970s. The sensitivity of CATT on blood is about 91% and its specificity is around 97%, making false positives an issue. Two field approaches for parasite detection—Mini Anion Exchange Centrifugation Technique (mAECT) and Capillary Tube Centrifugation (CTC)—also lack sensitivity (with mAECT achieving around 77% sensitivity and CTC around 56% sensitivity). For disease staging, a painful lumbar puncture is required to extract cerebrospinal fluid. The WHO criteria for a late-stage diagnosis is the presence of trypanosomes in cerebrospinal fluid or a white blood cell count of more than five cells per μL, or both [25]. Due to the complex and labour-intensive nature of HAT diagnosis, it has often been done by specialised mobile teams—engaged in active case finding—or in dedicated hospital settings.

Historically, innovation around drugs and diagnostics has not been not been a priority for HAT, and it is only recently with the advent of Product Development Partnerships that new tools have been developed [41]. Efforts to reach the 2020 elimination goal for HAT are on course. However, it is significant that the first phase of the campaign has been run vertically. To achieve the goal of zero transmission goal by 2030, HAT services will need to be integrated into public healthcare systems. While new tools (e.g., rapid diagnostic tests and new oral drug fexinidazole) have contributed to the idea that HAT services can be integrated [42], the placement of these tools in the lower tiers of health systems could yet prove challenging. The literature has already begun to point to some of the challenges that might emerge as weak health systems attempt to integrate HAT diagnosis [43,44].

The years covered by the scoping review coincide with a highly productive period for HAT diagnostic development, with the review papers documenting the development and validation of “first” generation (based on native antigens) and “second’ generation” (based on recombinant antigens) rapid diagnostic tests for screening [45,46,47,48,49,50,51,52,53,54,55]. Two “first” generation tests are already commercially available (Standard Diagnostics, Alere, Suwan, S. Korea; Coris Bioconcept, Gembloux, Belguim).

Different diagnostic approaches were also explored for parasitological detection [56,57,58], disease staging [54,59,60,61], and treatment follow-up [62,63]. A number of papers explored the potential of molecular tools for HAT, with many determining that LAMP displayed the greatest field potential at the current time [64,65,66,67,68,69].

Despite the great interest in generating new tools and testing approaches for HAT, it is significant that new diagnostic tools have so far done little to simplify the diagnostic tree for HAT (although the new drug fexinidazole should eventually remove the need for disease staging) [25]. Subsequently, a large portion of the literature is concerned with how best to deploy existing tools in support of elimination goals. In this respect, a lot of attention was assigned to screening approaches [43,44,70,71,72,73,74,75,76,77] and exploring testing algorithms [50,55].

While there is still room to improve HAT diagnosis, the tools that already exist are seen as “adequate” for securing elimination in the newest “NTD Roadmap”, requiring only some “modifications” to reach the 2030 target [2] (p. 34). That said, an aspiration for a new simplified and field-adapted diagnostic tool (one that does not require confirmatory testing by microscopy) is reported.

#### 3.2.2. Onchocerciasis

Onchocerciasis is a chronic parasitic disease caused by the filarial worm, Onchocerca volvulus, and is transmitted through bites of infected (Simulium) blackflies. It is the second leading cause of preventable blindness worldwide. Ivermectin is the main treatment for onchocerciasis. While it has a good activity against microfilariae, it does not kill adult O. volvulus worms, which can go on to repopulate microfilariae several months after treatment. Nevertheless community-directed treatment with ivermectin (CDTI) (typically once per year) is deemed an effective strategy for reducing microfilariae prevalence and the concentration of microfilariae in the target organs (skin and eyes) [78].

The impact of African Programme for Onchocerciasis Control (now replaced by the Expanded Special Project for Elimination of NTDs) was impressive, reducing disability-adjusted life year losses by 80% in foci countries [79]. However, such gains could yet be lost if CDTI is discontinued prematurely. The need to guide decision-making with regard to treatment has therefore created multiple entry points for diagnostics to help secure elimination: for mapping, mid-course monitoring and evaluation, determining where transmission has been interrupted (i.e., for stop-MDA decisions), and post-CDTI surveillance [78]. Elimination goals for onchocerciasis are presented in Box 3.

Box 3 **Elimination goals for onchocerciasis.**

**Goal: Regional Elimination**

The Onchocerciasis Elimination Program of the Americas (OEPA) began in 1992 with the goal of elimination at the outset. The target date for elimination of onchocerciasis transmission in the Region has been set as 2022. Four of the six endemic countries have already achieved elimination.

In 2012, WHO’s “NTD Roadmap” set a goal of elimination where feasible by 2020. The African Programme for Onchocerciasis Control advanced the goal to elimination in 80% of countries by 2025.

The tests and strategies that once proved useful for identifying priority areas for onchocerciasis interventions are unlikely to prove adequate for meeting elimination goals [78]. For instance, clinical examination has low sensitivity in areas of low prevalence, while the diethylcarbamazine (DEC) patch test and skin snip microscopy both lack sensitivity after treatment with ivermectin (which reduces the number of microfilariae in the skin to zero or near to zero). Furthermore, there is no point-of-care diagnostic that can distinguish between active and past infection, nor is there a test that can detect fecund adult female parasites in the human population (which could restart reproduction once MDA is stopped). Subsequently, a major focus of the literature centres on how diagnostics might support the four functions determined by the elimination goal. At point-of-care, antibody testing shows promise for supporting elimination programmes where the prevalence is high. ELISAs incorporating the Ov16 antigen are already widely used in elimination campaigns, while two rapid diagnostic tests based on Ov16 have become commercially available: a monoplex and a biplex for onchocerciasis and lymphatic filariasis (Standard Diagnostics, Suwan, S. Korea). However, WHO guidelines recommend further evaluation of the rapid diagnostic tests prior to their use in stop-MDA assessments [80].

In order to verify elimination, more sensitive and specific testing approaches are still required [39,78,81,82,83], and could—in theory—be achieved using a combination of tests [39].

Many papers sought to explore new testing approaches or ways to incrementally improve current methods [39,84,85,86,87,88,89,90,91]. In addition, common practice and official guidance were questioned [39,78,92].

The general consensus to emerge from the review is that the diagnostic arsenal for onchocerciasis is deficient. This aligns with the revised “NTD Roadmap”, which determines that existing tools for onchocerciasis require “major modifications” or might be considered “inadequate to reach 2030 targets” [2] (p. 34). As a first point of call, WHO and its partners would like to see the Ov16 monoplex and biplex onchocerciasis/lymphatic filariasis tests optimised, and a confirmatory diagnostic developed for use in low-prevalence settings to support a range of use cases.

#### 3.2.3. Schistosomiasis

Schistosomiasis, also known as bilharzia, is caused by blood flukes (trematode worms) of the genus Schistosoma. Infective larvae grow in an intermediate host (fresh-water snails) before penetrating the skin of the human host. Mature adult worms reside in the mesenteric (*S. mansoni* and *S. japonicum*) or pelvic (*S. haematobium*) veins, where female worms lay eggs that are later secreted in stools or urine. Eggs trapped in the surrounding tissues and organs cause inflammatory immune responses that result in intestinal, hepato-splenic, or urogenital disease [93]. Seventy-eight countries are endemic for schistosome infections, with different species of schistosomes affecting different regions. *S. haematobium* and *S. mansoni* are the main causative agents. The distribution of schistosomiasis is highly focal due to the fact that transmission relies on specific intermediary hosts and activities that expose humans to infection [94]. Elimination goals for schistosomiasis are presented in Box 4.

Box 4 **Elimination goals for schistosomiasis**

**Goal: Regional Elimination**

2015 regional elimination goals apply to the Eastern Mediterranean region, the Caribbean, Indonesia and the Mekong River Basin.

2020 regional elimination goals apply to the Region of the Americas, the Western Pacific Region and in selected countries in the African region.

Schistosomiasis demonstrates the complications that can reside behind an ostensibly simple MDA-based programme. The drug praziquantel is largely donated and is being administered in vast quantities, but the drug is not perfect; there are concerns around drug resistance, unpleasant side effects can impede uptake, and reinfection following treatment is swift. Diagnosis has therefore come to play a central role in better targeting elimination programmes that are being deployed at the community, individual, and environmental levels to assess reductions in disease, infections, and parasite transmission [95]. However, there are no simple diagnostic solutions.

The mainstay of schistosomiasis diagnosis is based on the detection of parasite eggs in urine or in faeces, for instance using urine filtration or examining faecal smears prepared by the Kato–Katz technique (microhaematuria has also been used as a proxy for urogenital schistosomiasis in some settings). However, because the number of excreted eggs is often low and shows much day-to-day fluctuation, these methods are not considered sensitive enough to support elimination goals in low-endemicity settings. Serology—the testing of antibody responses—provides high levels of sensitivity and specificity but does not disclose the intensity of infection, and cannot differentiate between active and past infection. To achieve even higher sensitivity and identify active infection, research has concentrated on the amplification of pathogen DNA using techniques based on polymerase chain reaction or LAMP. High sensitivities capable of determining active infection have also been achieved with assays for the detection of circulating anodic antigen (CAA) and circulating cathodic antigen (CCA) in serum or urine [96], including a commercially available CCA test (Rapid Medical Diagnostics, Pretoria, South Africa).

A number of papers reviewed the available diagnostic approaches for schistosomiasis, finding room for improvement with each [95,96,97,98,99,100,101]. Subsequently, the emerging consensus is for a stratified approach, by which difference test combinations are deployed for different use cases and tailored to the exigencies of national programmes [96,102,103,104,105,106]. In this context, the correct question for schistosomiasis elimination is not “What is the best tool?” but “What are the best diagnostic methods?” and how these apply to each of the stages of control and elimination [96,102]. Several papers set out to address these questions through the development of Target Product Profiles [8,106,107,108].

According to the new “NTD Roadmap”, although diagnostic tools to support the elimination of schistosomiasis already exist, across the board they are require “major modifications” or are deemed “inadequate to reach 2030 targets” [2] (p. 34). Ideally, WHO and its partners would like to see the development of a standardised, sensitive, point-of-care diagnostic for use in various prevalence settings and for all schistosome species.

## 4. Discussion

Where once the role of diagnosis in guiding clinical decision-making in relation to individual patients was assumed (and conceptualised as linear), today a wealth of literature exists to challenge this position. Not only is the idea that diagnosis informs treatment pathways questionable, but the notion that diagnosis is something that happens to an individual is changing. The exigencies of disease NTD elimination programmes exemplify both shifts. Individuals may still retain their central position at the heart of IDM programmes like HAT and visceral leishmaniasis, but for diseases amendable to MDA, like schistosomiasis or onchocerciasis, the focus of diagnosis is currently on the collective. Furthermore, a positive diagnosis may not result in access to treatment if programmatic thresholds are not met. Yet, the IDM/MDA dichotomy is not set in stone and could shift as new tools are developed or if MDA programmes move to test and treat strategies.

In support of elimination goals, the proposed uses of diagnostics have grown. For IDM diseases, the foremost need is for confirmatory diagnosis, but in theory other functions would support disease programmes—tests of drug efficacy, tests of cure, and tests capable of picking up emerging drug resistance. For MDA-amendable diseases, there are at least four entry points (termed use cases) for diagnosis: disease mapping, impact monitoring, stop-MDA decision-making, and surveillance response. However, assays testing drug efficacy and looking for resistance would be helpful add-ons.

While initially the yield of included studies was sub-divided into two pots for practical purposes, one consequence of synthesising the results in this way has been to reveal a disconnect between the speculative nature of debate in the “global overview” papers—which to some extent assume that better diagnostics are both needed and coming—and the messy reality of the testing approaches disease programmes are currently utilising. This is well exemplified for the post-elimination scenario in relation to the surveillance and response systems. Here, we are told, programmes will need testing approaches that are sufficiently specific and sensitive, preferably supported by mobile and electronic technologies, and—for IDM diseases—deployable at the point-of-care. Yet, the review of diagnostic needs “by disease” suggests we are very far from this vision, and this is confirmed by the overview provided by WHO in the new “NTD Roadmap” [2]. Disease programmes are attempting to further elimination goals with less than perfect diagnostic approaches. There are few “gold standards”. In a bid to address the many deficiencies and unknowns, programmes might choose to conduct repeat testing and/or deploy tests in combinations and algorithms. However, official guidance over which tests to deploy, when, and in what combination is not always available and can be subject to change. The use of the humble microscope and skilled technician retains a centrally important role in this mix.

Diagnosis appears to offer certainty to disease programmes (and the donors supporting them) as they approach elimination goals, but this review speaks largely to uncertainty. What is meant by disease elimination varies by disease; the distinction between IDM and MDA-amendable NTDs could shift if the right tool was devised; mapping approaches, sampling frames, and thresholds for starting MDA are all subject to revision; epidemiological knowledge is known to be wanting; and tests that perform well in the lab for unknown reasons fail in the field. One response to dealing with such uncertainty is to declare a need for new technologies. Not just new and better tests, but technology that could support existing approaches by transmitting, storing, and analysing diagnostic data; and to quality check results (RDT readers, video of microscope views). Where the funding might be found to support product innovation in the field of diagnostics is unclear at this time.

A lot of the research picked up in the review related to basic research or tools and approaches with little field potential. This feels like a missed opportunity. The review did pick up draft Target Product Profiles for NTDs like Chagas disease and schistosomiasis [8,106,108,109]. Target Product Profiles could and should play a role in aligning research to real world uses to maximise the utility of limited resources, but only if they are informed by the patients who need them and the health systems that will use them. As Tambo et al. have stated, “a deeper understanding of which tools and strategies are most suitable to achieve elimination…are needed” [29] (p. 8).

## 5. Conclusions

This review has upheld the growing consensus that diagnostic technologies will need to play a pivotal role in determining the success of NTD elimination goals. Yet, it has also concluded that there remains substantial work to do. To drive research and attract investment, the field of diagnosis for NTD elimination urgently needs a guiding framework. It is pertinent, therefore, that in 2019 the WHO convened a Diagnostic Technical Advisory Group to assess and prioritise diagnostic needs for the portfolio of NTDs the WHO supports. As part of its work, the group will review existing Target Product Profiles and support the development of new ones wherever critical gaps are identified [110].

Just as early NTD campaigning set about drumming up support for MDA campaigns [10], now donors (including pharmaceutical companies) will need to be convinced of the merit of supporting the deployment, refinement, and development of diagnostic technologies in support of NTD elimination goals. The argument is actually very compelling; diagnostics in every use case are a means of better targeting and thus rationalising resources. Moreover, they will play a central role in sustaining programmatic successes in relation to disease elimination, which will require surveillance and response systems that can point to new and re-emerging infections. The incorporation of several NTD tests in the second edition of the Essential Diagnostics List in 2019 was a useful first step toward building this messaging [111].

## Figures and Tables

**Table 1:** Neglected Tropical Disease (NTD) elimination targets [3] (p. 19).

Disease	Target
Chagas Disease	Regional elimination
Human African Trypanosomiasis	Global elimination
Human Rabies	Regional elimination
Leprosy	Global elimination
Lymphatic Filariasis	Regional elimination
Onchocerciasis	Regional elimination
Schistosomiasis	Regional elimination
Trachoma	Global elimination
Visceral Leishmaniasis	Global elimination

**Table 2:** Scoping review results in aggregate and by disease.

	Initial Search Yield (*n* =)	Included Papers (*n* =)
Chagas disease	383	45
Human African trypanosomiasis	1271	106
Lymphatic filariasis	1527	145
Onchocerciasis	59	35
Schistosomiasis	117	78
Trachoma	28	16
Visceral leishmaniasis	80	44
Minus duplicates		20
TOTAL	3465	468−20 = 448

## Supplementary Materials

The following are available online at https://www.mdpi.com/2075-4418/10/6/375/s1, Table S1: NTDiagnostics_Included studies; File S1: Overview WoS results_search strings, dates and numbers.

## References

[1] Kohli M., Sen P., Pai M. (2019). Improving access to essential tests for infectious diseases. Microbes Infect..

[2] WHO (2020). Ending the Neglect to Attain the SDGs: A Roadmap for Neglected Tropical Diseases 2021–2030 Geneva. https://www.who.int/neglected_diseases/Ending-the-neglect-to-attain-the-SDGs--NTD-Roadmap.pdf?ua=1.

[3] WHO (2012). Accelerating Work to Overcome the Global Impact of Neglected Tropical Diseases: A Roadmap for Implementation Geneva. https://www.who.int/neglected_diseases/NTD_RoadMap_2012_Fullversion.pdf.

[4] WHO Water Sanitation and Hygiene for Accelerating and Sustaining Progress on Neglected Tropical Diseases: A Global Strategy 2015–2020. https://www.who.int/water_sanitation_health/publications/wash-and-ntd-strategy/en/.

[5] WHO Global Vector Control Response 2017–2030. https://www.who.int/vector-control/publications/global-control-response/en/.

[6] ESPEN Collect Platform. http://espen.afro.who.int/tools-resources/espen-collect.

[7] Tropical Data. https://www.tropicaldata.

[8] Solomon A.W., Engels D., Bailey R.L., Blake I.M., Brooker S., Chen J.-X., Chen J.-H., Churcher T.S., Drakeley C.J., Edwards T. (2012). A Diagnostics Platform for the Integrated Mapping, Monitoring, and Surveillance of Neglected Tropical Diseases: Rationale and Target Product Profiles. PLoS Negl. Trop. Dis..

[9] Smith J., Taylor E.M. (2013). MDGs and NTDs: Reshaping the global health agenda. PLoS Negl. Trop. Dis..

[10] Molyneux D.H., Hotez P.J., Fenwick A. (2005). “Rapid-Impact Interventions”: How a Policy of Integrated Control for Africa’s Neglected Tropical Diseases Could Benefit the Poor. PLoS Med..

[11] Molyneux D.H. (2012). The “Neglected Tropical Diseases”: Now a brand identity; Responsibilities, context and promise. Parasites Vectors.

[12] WHO (2010). Working to Overcome the Global Impact of Neglected Tropical Diseases: First WHO Report on Neglected Tropical Diseases.

[13] Uniting to Combat Neglected Tropical Diseases. London Declaration on Neglected Tropical Diseases. https://www.who.int/neglected_diseases/London_Declaration_NTDs.pdf.

[14] Taylor E.M., Smith J. (2018). Neglected Tropical Diseases and Equity in the Post-2015 Health Agenda. IDS Bull..

[15] Smith J., Taylor E.M. (2016). What Is Next for NTDs in the Era of the Sustainable Development Goals?. PLoS Negl. Trop. Dis..

[16] Stepan N. (2011). Eradication: Ridding the World of Diseases Forever?.

[17] CDC Recommendation of the International Taskforce for Disease Eradication 2008. https://www.cartercenter.org/resources/pdfs/news/health_publications/itfde/updated_disease_candidate_table.pdf.

[18] Molyneux D.H., Hopkins D.R., Zagaria N. (2004). Disease eradication, elimination and control: The need for accurate and consistent usage. Trends Parasitol..

[19] Hopkins D.R. (2013). Disease eradication. New Engl. J. Med..

[20] Dowdle W.R., Cochi S.L. The Principles and Feasibility of Disease Eradication. http://www.ncbi.nlm.nih.gov/pubmed/22188936.

[21] Hotez P.J., Pécoul B., Rijal S., Boehme C., Aksoy S., Malecela M., Tapia-Conyer R., Reeder J. (2016). Eliminating the neglected tropical diseases: Translational science and new technologies. PLoS Negl. Trop. Dis..

[22] Hollingsworth T.D. (2018). Counting Down the 2020 Goals for 9 Neglected Tropical Diseases: What Have We Learned From Quantitative Analysis and Transmission Modeling?. Clin. Infect. Dis..

[23] Jervis S., Chapman L.A.C., Dwivedi S., Karthick M., Das A., Le Rutte E.A., Courtenay O., Medley G.F., Banerjee I., Mahapatra T. (2017). Variations in visceral leishmaniasis burden, mortality and the pathway to care within Bihar, India. Parasites Vectors.

[24] Peeling R.W., Mabey D.C. (2014). Diagnostics for the control and elimination of neglected tropical diseases. Parasitology.

[25] Bonnet J., Boudot C., Courtioux B. (2015). Overview of the diagnostic methods used in the field for human african trypanosomiasis: What could change in the next years?. BioMed Res. Int..

[26] Peeling R.W. (2015). Diagnostics in a digital age: An opportunity to strengthen health systems and improve health outcomes. Int. Heal..

[27] Albonico M., Levecke B., LoVerde P., Montresor A., Prichard R., Vercruysse J., Webster J. (2015). Monitoring the efficacy of drugs for neglected tropical diseases controlled by preventive chemotherapy. J. Glob. Antimicrob. Resist..

[28] Zhou X.-N., Bergquist R., Tanner M. (2013). Elimination of tropical disease through surveillance and response. Infect. Dis. Poverty.

[29] Ai L., Zhou X., Chen J.-H., Hu W., Bergquist R., Guo J.-G., Utzinger J., Tanner M., Zhou X.-N., E1T (2014). Surveillance-response systems: The key to elimination of tropical diseases. Infect. Dis. Poverty.

[30] Bergquist R., Yang G.-J., Knopp S., Utzinger J., Tanner M. (2015). Surveillance and response: Tools and approaches for the elimination stage of neglected tropical diseases. Acta Trop..

[31] Utzinger J., Becker S.L., Knopp S., Blum J., Neumayr A., Keiser J., Hatz C. (2012). Neglected tropical diseases: Diagnosis, clinical management, treatment and control. Swiss Med. Wkly..

[32] Pion S.D., Montavon C., Chesnais C.B., Kamgno J., Wanji S., Klion A.D., Nutman T.B., Boussinesq M. (2016). Positivity of Antigen Tests Used for Diagnosis of Lymphatic Filariasis in Individuals Without *Wuchereria bancrofti* Infection But with High *Loa loa* Microfilaremia. Am. J. Trop. Med. Hyg..

[33] Wanji S., Amvongo-Adjia N., Koudou B., Njouendou A.J., Ndongmo P.W.C., Kengne-Ouafo J.A., Datchoua-Poutcheu F.R., Fovennso B.A., Tayong D.B., Fombad F.F. (2015). Cross-Reactivity of Filariais ICT Cards in Areas of Contrasting Endemicity of *Loa loa* and *Mansonella perstans* in Cameroon: Implications for Shrinking of the Lymphatic Filariasis Map in the Central African Region. PLoS Negl. Trop. Dis..

[34] Stanton M.C., Mkwanda S.Z., Debrah A., Debrah L.B., Biritwum N.-K., Hoerauf A., Cliffe M., Best A., Molineux A., Kelly-Hope L.A. (2015). Developing a community-led SMS reporting tool for the rapid assessment of lymphatic filariasis morbidity burden: Case studies from Malawi and Ghana. BMC Infect. Dis..

[35] Gurunath U., Joshi R., Agrawal A., Shah V. (2014). An overview of visceral leishmaniasis elimination program in India: A picture imperfect. Expert Rev. Anti Infect. Ther..

[36] Peeling R.W., Boeras D.I., Nkengasong J. (2017). Re-imagining the future of diagnosis of Neglected Tropical Diseases. Comput. Struct. Biotechnol. J..

[37] Lammie P.J., Moss D.M., Goodhew B., Hamlin K., Krolewiecki A., West S.K., Priest J.W. (2012). Development of a new platform for neglected tropical disease surveillance. Int. J. Parasitol..

[38] Steel C., Golden A., Stevens E., Yokobe L., Domingo G.J., Santos T.D.L., Nutman T.B. (2015). Rapid Point-of-Contact Tool for Mapping and Integrated Surveillance of *Wuchereria bancrofti* and *Onchocerca volvulus* Infection. Clin. Vaccine Immunol..

[39] Unnasch T.R., Golden A., Cama V., Cantey P.T. (2018). Diagnostics for onchocerciasis in the era of elimination. Int. Heal..

[40] Ndung J.M., Bieler S., Roscigno G. (2010). “Piggy-Backing” on Diagnostic Platforms Brings Hope to Neglected Diseases: The Case of Sleeping Sickness. PLoS Negl. Trop. Dis..

[41] Taylor E.M., Smith J. (2020). Product Development Partnerships: Delivering Innovation for the Elimination of African Trypanosomiasis?. Trop. Med. Infect. Dis..

[42] Franco J.R., Simarro P.P., Diarra A., Ruiz-Postigo J.A., Jannin J.G. (2014). The journey towards elimination of gambiense human African trypanosomiasis: Not far, nor easy. Parasitology.

[43] Mitashi P., Hasker E., Mbo F., Van Geertruyden J.-P., Kaswa M., Lumbala C., Boelaert M., Lutumba P. (2014). Integration of diagnosis and treatment of sleeping sickness in primary healthcare facilities in the Democratic Republic of the Congo. Trop. Med. Int. Heal..

[44] Lee S.J., Palmer J. (2018). Integrating innovations: A qualitative analysis of referral non-completion among rapid diagnostic test-positive patients in Uganda’s human African trypanosomiasis elimination programme. Infect. Dis. Poverty.

[45] Sullivan L., Wall S.J., Carrington M., Ferguson M.A.J. (2013). Proteomic Selection of Immunodiagnostic Antigens for Human African Trypanosomiasis and Generation of a Prototype Lateral Flow Immunodiagnostic Device. PLoS Negl. Trop. Dis..

[46] Büscher P., Gilleman Q., Lejon V. (2013). Rapid Diagnostic Test for Sleeping Sickness. New Engl. J. Med..

[47] Büscher P., Mertens P., Leclipteux T., Gilleman Q., Jacquet D., Mumba-Ngoyi D., Pyana P.P., Boelaert M., Lejon V. (2014). Sensitivity and specificity of HAT Sero-K-SeT, a rapid diagnostic test for serodiagnosis of sleeping sickness caused by *Trypanosoma brucei gambiense*: A case-control study. Lancet Glob. Heal..

[48] Boelaert M., Mukendi D., Bottieau E., Lilo J.R.K., Verdonck K., Minikulu L., Barbé B., Gillet P., Yansouni C.P., Chappuis F. (2018). A Phase III Diagnostic Accuracy Study of a Rapid Diagnostic Test for Diagnosis of Second-Stage Human African Trypanosomiasis in the Democratic Republic of the Congo. EBioMedicine.

[49] Bisser S., Lumbala C., Nguertoum E., Kande V., Flevaud L., Vatunga G., Boelaert M., Büscher P., Josenando T., Bessell P.R. (2016). Sensitivity and Specificity of a Prototype Rapid Diagnostic Test for the Detection of *Trypanosoma brucei gambiense* Infection: A Multi-centric Prospective Study. PLoS Negl. Trop. Dis..

[50] Lumbala C., Bessell P.R., Lutumba P., Baloji S., Bieler S., Ndung J.M. (2017). Performance of the SD BIOLINE^®^ HAT rapid test in various diagnostic algorithms for gambiense human African trypanosomiasis in the Democratic Republic of the Congo. PLoS ONE.

[51] Van Nieuwenhove L., Büscher P., Balharbi F., Humbert M., Dieltjens T., Guisez Y., Lejon V. (2012). Identification of Mimotopes with Diagnostic Potential for *Trypanosoma brucei gambiense* Variant Surface Glycoproteins Using Human Antibody Fractions. PLoS Neglected Trop. Dis..

[52] Sullivan L., Fleming J., Sastry L., Mehlert A., Wall S.J., Ferguson M.A.J. (2014). Identification of sVSG117 as an Immunodiagnostic Antigen and Evaluation of a Dual-Antigen Lateral Flow Test for the Diagnosis of Human African Trypanosomiasis. PLoS Neglected Trop. Dis..

[53] Rogé S., Van Nieuwenhove L., Meul M., Heykers A., De Koning A.B., Bebronne N., Guisez Y., Büscher P. (2014). Recombinant Antigens Expressed in *Pichia pastoris* for the Diagnosis of Sleeping Sickness Caused by *Trypanosoma brucei* gambiense. PLoS Negl. Trop. Dis..

[54] Sternberg J., Mitchell J.A. (2014). Plasma neuronal specific enolase: A potential stage diagnostic marker in human African trypanosomiasis. Trans. R. Soc. Trop. Med. Hyg..

[55] Lumbala C., Biéler S., Kayembe S., Makabuza J., Ongarello S., Ndung’U J.M. (2018). Prospective evaluation of a rapid diagnostic test for *Trypanosoma brucei gambiense* infection developed using recombinant antigens. PLoS Negl. Trop. Dis..

[56] Bieler S., Matovu E., Mitashi P., Ssewannyana E., Shamamba S.K.B., Bessell P.R., Ndung J.M. (2012). Improved detection of *Trypanosoma brucei* by lysis of red blood cells, concentration and LED fluorescence microscopy. Acta Trop..

[57] Giordani F., Munde M., Wilson W.D., Ismail M.A., Kumar A., Boykin D.W., Barrett M.P. (2013). Green Fluorescent Diamidines as Diagnostic Probes for Trypanosomes. Antimicrob. Agents Chemother..

[58] Ngoyi D.M., Ekangu R.A., Kodi M.F.M., Pyana P.P., Balharbi F., Decq M., Betu V.K., Van Der Veken W., Sese C., Menten J. (2014). Performance of Parasitological and Molecular Techniques for the Diagnosis and Surveillance of Gambiense Sleeping Sickness. PLoS Negl. Trop. Dis..

[59] Burchmore R. (2012). Parasites in the brain? The search for sleeping sickness biomarkers. Expert Rev. Anti Infect. Ther..

[60] Ngoyi D.M., Menten J., Pyana P.P., Büscher P., Lejon V. (2013). Stage determination in sleeping sickness: Comparison of two cell counting and two parasite detection techniques. Trop. Med. Int. Heal..

[61] Abdulla M.-H., Bakhiet M., Lejon V., Andersson J., McKerrow J., Al-Obeed O., Harris R.A. (2013). TLTF in Cerebrospinal Fluid for Detection and Staging of *T. b. gambiense* Infection. PLoS ONE.

[62] Tiberti N., Lejon V., Hainard A., Courtioux B., Robin X., Turck N., Kristensson K., Matovu E., Enyaru J.C., Ngoyi D.M. (2013). Neopterin is a cerebrospinal fluid marker for treatment outcome evaluation in patients affected by *Trypanosoma brucei gambiense* sleeping sickness. PLoS Neglected Trop. Dis..

[63] Ilboudo H., Camara O., Ravel S., Bucheton B., Lejon V., Camara M., Kaboré J., Jamonneau V., Deborggraeve S. (2015). The trypanosome’s spliced leader RNA is a more specific marker for cure of human African trypanosomiasis than DNA. J. Infect. Dis..

[64] Deborggraeve S., Büscher P. (2012). Recent progress in molecular diagnosis of sleeping sickness. Expert Rev. Mol. Diagn..

[65] Büscher P., Deborggraeve S. (2015). How can molecular diagnostics contribute to the elimination of human African trypanosomiasis?. Expert Rev. Mol. Diagn..

[66] Namangala B., Hachaambwa L., Kajino K., Mweene A.S., Hayashida K., Simuunza M., Simukoko H., Choongo K., Chansa P., Lakhi S. (2012). The use of Loop-mediated Isothermal Amplification (LAMP) to detect the re-emerging Human African Trypanosomiasis (HAT) in the Luangwa and Zambezi valleys. Parasites Vectors.

[67] Mitashi P., Hasker E., Ngoyi D.M., Pyana P.P., Lejon V., Van Der Veken W., Lutumba P., Büscher P., Boelaert M., Deborggraeve S. (2013). Diagnostic Accuracy of Loopamp *Trypanosoma brucei* Detection Kit for Diagnosis of Human African Trypanosomiasis in Clinical Samples. PLoS Negl. Trop. Dis..

[68] Hayashida K., Kajino K., Hachaambwa L., Namangala B., Sugimoto C. (2015). Direct Blood Dry LAMP: A Rapid, Stable, and Easy Diagnostic Tool for Human African Trypanosomiasis. PLoS Negl. Trop. Dis..

[69] Nikolskaia O.V., Thekisoe O., Dumler J.S., Grab D.J. (2016). Loop-Mediated Isothermal Amplification for Detection of the 5.8S Ribosomal Ribonucleic Acid Internal Transcribed Spacer 2 Gene Found in *Trypanosoma brucei gambiense*. Am. J. Trop. Med. Hyg..

[70] Rock K.S., Torr S.J., Lumbala C., Keeling M. (2015). Quantitative evaluation of the strategy to eliminate human African trypanosomiasis in the Democratic Republic of Congo. Parasites Vectors.

[71] Mathurin K., Djetchi M., Ilboudo H., Kaba D., Coulibaly B., Gouan E., Kouakou L., Bucheton B., Solano P., Courtin F. (2016). A targeted door-to-door strategy for sleeping sickness detection in low-prevalence settings in Côte d’Ivoire. Parasite.

[72] Checchi F., Cox A.P., Chappuis F., Priotto G., Chandramohan D., Haydon D.T. (2012). Prevalence and under-detection of gambiense human African trypanosomiasis during mass screening sessions in Uganda and Sudan. Parasites Vectors.

[73] Lejon V., Jacobs J., Simarro P.P. (2013). Elimination of sleeping sickness hindered by difficult diagnosis. Bull. World Heal. Organ..

[74] Simarro P., Cecchi G., Franco J.R., Paone M., Diarra A., Postigo J.A.R., Mattioli R.C., Jannin J.G. (2014). Mapping the capacities of fixed health facilities to cover people at risk of gambiense human African trypanosomiasis. Int. J. Heal. Geogr..

[75] Wamboga C., Matovu E., Bessell P.R., Picado A., Bieler S., Ndung J.M. (2017). Enhanced passive screening and diagnosis for gambiense human African trypanosomiasis in north-western Uganda—Moving towards elimination. PLoS ONE.

[76] Checchi F., Funk S., Chandramohan D., Chappuis F., Haydon D.T. (2018). The impact of passive case detection on the transmission dynamics of gambiense Human African Trypanosomiasis. PLoS Negl. Trop. Dis..

[77] Palmer J., Robert O., Kansiime F. (2017). Including refugees in disease elimination: Challenges observed from a sleeping sickness programme in Uganda. Confl. Heal..

[78] Vlaminck J., Fischer P.U., Weil G.J. (2015). Diagnostic Tools for Onchocerciasis Elimination Programs. Trends Parasitol..

[79] Coffeng L.E., Stolk W.A., Zouré H.G.M., Veerman L., Agblewonu K.B., Murdoch M.E., Noma M., Fobi G., Richardus J.H., Bundy D.A.P. (2014). African Programme for Onchocerciasis Control 1995–2015: Updated Health Impact Estimates Based on New Disability Weights. PLoS Neglected Trop. Dis..

[80] WHO/Department of Control of Neglected Tropical Diseases Guidelines for Stopping Mass Drug Administration and Verifying Elimination of Human Onchocerciasis: Criteria and Procedures. https://www.who.int/onchocerciasis/resources/9789241510011/en/.

[81] Golden A., Faulx D., Kalnoky M., Stevens E., Yokobe L., Peck R., Karabou P., Banla M., Rao R., Adade K. (2016). Analysis of age-dependent trends in Ov16 IgG4 seroprevalence to onchocerciasis. Parasites Vectors.

[82] Lont Y.L., Coffeng L.E., De Vlas S.J., Golden A., Santos T.D.L., Domingo G.J., Stolk W. (2017). Modelling Anti-Ov16 IgG4 Antibody Prevalence as an Indicator for Evaluation and Decision Making in Onchocerciasis Elimination Programmes. PLoS Negl. Trop. Dis..

[83] Dieye Y., Storey H.L., Barrett K.L., Gerth-Guyette E., Di Giorgio L., Golden A., Faulx D., Kalnoky M., Ndiaye M.K.N., Sy N. (2017). Feasibility of utilizing the SD BIOLINE Onchocerciasis IgG4 rapid test in onchocerciasis surveillance in Senegal. PLoS Negl. Trop. Dis..

[84] Globisch D., Moreno A.Y., Hixon M.S., Nunes A.A.K., Denery J.R., Specht S., Hoerauf A., Janda K.D. (2013). *Onchocerca volvulus*-neurotransmitter tyramine is a biomarker for river blindness. Proc. Natl. Acad. Sci. USA.

[85] Shirey R.J., Globisch D., Eubanks L.M., Hixon M.S., Janda K.D. (2018). Noninvasive Urine Biomarker Lateral Flow Immunoassay for Monitoring Active Onchocerciasis. ACS Infect. Dis..

[86] Lloyd M.M., Gilbert R., Taha N.T., Weil G.J., Meïté A., Kouakou I.M., Fischer P.U., Taha T.Y.N. (2015). Conventional parasitology and DNA-based diagnostic methods for onchocerciasis elimination programmes. Acta Trop..

[87] Mekonnen S.A., Beissner M., Saar M., Ali S., Zeynudin A., Kassahun T., Adbaru M.G., Battke F., Poppert S., Hoelscher M. (2017). O-5S quantitative real-time PCR: A new diagnostic tool for laboratory confirmation of human onchocerciasis. Parasites Vectors.

[88] Prince-Guerra J.L., Cama V.A., Wilson N., Thiele E.A., Likwela J., Ndakala N., Muzinga J.M.W., Ayebazibwe N., Ndjakani Y.D., Pitchouna N.A. (2018). Comparison of PCR Methods for *Onchocerca volvulus* Detection in Skin Snip Biopsies from the Tshopo Province, Democratic Republic of the Congo. Am. J. Trop. Med. Hyg..

[89] Lagatie O., Merino M., Debrah L.B., Debrah A., Stuyver L. (2016). An isothermal DNA amplification method for detection of *Onchocerca volvulus* infection in skin biopsies. Parasites Vectors.

[90] Poole C.B., Li Z., Alhassan A., Guelig D., Diesburg S., Tanner N.A., Zhang Y., Evans T.C., Labarre P., Wanji S. (2017). Colorimetric tests for diagnosis of filarial infection and vector surveillance using non-instrumented nucleic acid loop-mediated isothermal amplification (NINA-LAMP). PLoS ONE.

[91] Golden A., Stevens E.J., Yokobe L., Faulx D., Kalnoky M., Peck R., Valdez M., Steel C., Karabou P., Banla M. (2016). A Recombinant Positive Control for Serology Diagnostic Tests Supporting Elimination of *Onchocerca volvulus*. PLoS Negl. Trop. Dis..

[92] Rebollo M.P., Zouré H., Ogoussan K., Sodahlon Y., A Ottesen E., Cantey P.T. (2018). Onchocerciasis: Shifting the target from control to elimination requires a new first-step—Elimination mapping. Int. Heal..

[93] Deng Y., Qiu C., Ding H., Lu D.-B. (2018). The ratio of the seroprevalence to the egg-positive prevalence of *Schistosoma japonicum* in China: A meta-analysis. BMC Infect. Dis..

[94] WHO/Department of Control of Neglected Tropical Diseases Integrating Neglected Tropical Diseases into Global Health and Development: Fourth WHO Report on Neglected Tropical Diseases. https://www.who.int/neglected_diseases/resources/9789241565448/en/.

[95] Stothard J.R., Stanton M.C., Bustinduy A.L., Sousa-Figueiredo J.C., Van Dam G.J., Betson M., Waterhouse D., Ward S., Allan F., Hassan A.A. (2014). Diagnostics for schistosomiasis in Africa and Arabia: A review of present options in control and future needs for elimination. Parasitology.

[96] Van Dam G.J., Xu J., Bergquist R., De Dood C., Utzinger J., Qin Z.-Q., Guan W., Feng T., Yu X.-L., Zhou J. (2015). An ultra-sensitive assay targeting the circulating anodic antigen for the diagnosis of *Schistosoma japonicum* in a low-endemic area, People’s Republic of China. Acta Trop..

[97] Le L., Hsieh M. (2017). Diagnosing Urogenital Schistosomiasis: Dealing with Diminishing Returns. Trends Parasitol..

[98] Ogongo P., Kariuki T.M., Wilson R.A. (2018). Diagnosis of *Schistosomiasis mansoni*: An evaluation of existing methods and research towards single worm pair detection. Parasitology.

[99] Weerakoon K.G., Gobert G.N., Cai P., McManus D.P. (2015). Advances in the Diagnosis of Human Schistosomiasis. Clin. Microbiol. Rev..

[100] Bergquist R., Zhou X.-N., Rollinson D., Reinhard-Rupp J., Klohe K. (2017). Elimination of schistosomiasis: The tools required. Infect. Dis. Poverty.

[101] Gomes L.I., Enk M.J., Rabello A. (2014). Diagnosing schistosomiasis: Where are we?. Rev. Soc. Bras. Med. Trop..

[102] Bergquist R., Johansen M.V., Utzinger J. (2009). Diagnostic dilemmas in helminthology: What tools to use and when?. Trends Parasitol..

[103] Zhang J.-F., Xu J., Bergquist R., Yu L.-L., Yan X.-L., Zhu H.-Q., Wen L.-Y. (2016). Development and application of diagnostics in the national schistosomiasis control programme in The People’s Republic of China. Advances in Parasitology.

[104] Belizario V., Bungay A.A., Su G.S., De Veyra C., Lacuna J.D. (2016). Assessment of three schistosomiasis endemic areas using kato-katz technique and elisa antigen and antibody tests. Southeast Asian J. Trop. Med. Public Heal..

[105] Etet P.F.S., Mahomoodally M.F. (2012). New Insights in Staging and Chemotherapy of African Trypanosomiasis and Possible Contribution of Medicinal Plants. Sci. World J..

[106] Lim M.D., Brooker S.J., Belizario V.Y., Gay-Andrieu F., Gilleard J.S., Levecke B., Van Lieshout L., Medley G., Mekonnen Z., Mirams G. (2018). Diagnostic tools for soil-transmitted helminths control and elimination programs: A pathway for diagnostic product development. PLoS Negl. Trop. Dis..

[107] Utzinger J., Becker S., Van Lieshout L., Van Dam G., Knopp S. (2015). New diagnostic tools in schistosomiasis. Clin. Microbiol. Infect..

[108] Hawkins K.R., Cantera J.L., Storey H.L., Leader B.T., Santos T.D.L. (2016). Diagnostic Tests to Support Late-Stage Control Programs for Schistosomiasis and Soil-Transmitted Helminthiases. PLoS Negl. Trop. Dis..

[109] Porrás A.I., Yadon Z.E., Altcheh J., Britto C., Chaves G.C., Flevaud L., Martins-Filho O.A., Ribeiro I., Schijman A.G., Shikanai-Yasuda M.A. (2015). Target Product Profile (TPP) for Chagas Disease Point-of-Care Diagnosis and Assessment of Response to Treatment. PLoS Negl. Trop. Dis..

[110] WHO Report of the First Meeting of the WHO Diagnostic Technical Advisory Group for Neglected Tropical Diseases. https://www.who.int/neglected_diseases/resources/9789240003590/en/.

[111] WHO Second WHO Model List of Essential In Vitro Diagnostics. https://www.who.int/medical_devices/publications/EDL_2_0_Standalone.pdf?ua=1.

