# Supplementary material for: NTD Diagnostics for Disease Elimination: A Review
Source: Diagnostics (Basel). 2020 Jun 5;10(6):375. doi: 10.3390/diagnostics10060375 (PMC7344624; doi:10.3390/diagnostics10060375)
Supplement: Supplementary file 1 [file diagnostics-10-00375-s001.zip › Supplementary/File S1 Overview WoS results_search strings, dates and numbers.docx]

**OVERVIEW: SEARCH STRINGS, DATES AND RESULTS**

*Basic searches conducted unless otherwise stated, then an advanced search on topic used*

**Web of Science Core Collection, searched 14 Jan 2019**

**American trypanosomiasis OR Chagas disease AND elimination AND diagnos* (years 2012-2018)**

**= 383 results,** of which 45 included in review

**Web of Science Core Collection, searched 19 Dec 2018**

**Human African Trypanosomiasis OR sleeping sickness AND elimination AND diagnos* (years 2012-2018)**

**= 1,271 results,** of which 106 included in review

**Web of Science Core Collection, searched 7 Jan 2019**

**Lymphatic Filariasis OR elephantiasis AND diagnos* AND elimination (years 2012-2018)**

**= 1,527 results,** of which 145 included in review

**Web of Science Core Collection, searched 14 Jan 2019**

First I attempted a general search for Oncho but the yield was unwieldy; i.e.

Onchocerciasis OR River blindness AND elimination AND diagnos* (years 2012-2018)

= 846 results

Then I conducted an advanced search based on topic, which matches the search string with the Abstract, Title, and/or Keywords fields of a record. Here I used

**TS=(onchocerciasis AND elimination AND diagnos*)**

Refined to timespan **2012-2018**.

**= 59 results**, of which 35 included in review

**Web of Science Core Collection, searched 14 Jan 2019**

First I attempted a basic search for schisto but the yield was too unwieldy; i.e.

Schistosomiasis OR Bilharza AND elimination AND diagnos* (years 2012-2018)

= 4,043 results

Instead I used an advanced search based on topic, which matches the search string with the Abstract, Title, and/or Keywords fields of a record. Here I used

**TS=(schistosom* AND elimination AND diagnos*)**

Refined to timespan **2012-2018**.

**= 117 results**, of which 78 included in review

**Web of Science Core Collection, searched 14 Jan 2019**

**Trachom* AND elimination AND diagnos* (years 2012-2018)**

**= 28 results**, of which 16 included in review

**Web of Science Core Collection, searched 14 Jan 2019**

First I attempted a basic search for VL but the yield was too unwieldy; i.e.

Visceral Leishmaniasis OR Kala-Azar AND diagnos* AND elimination (years 2012-2018)

= 5, 177

Instead I used an advanced search based on topic, which matches the search string with the Abstract, Title, and/or Keywords fields of a record. Here I used

**TS=(visceral leishmaniasis AND elimination AND diagnos*)**

Refined to timespan **2012-2018**.

**= 80 results**, of which 44 included in review
